# Non-union of Bone Plating in a Mandibular Fracture With Osteomyelitis: A Case Report

**DOI:** 10.7759/cureus.116282

**Published:** 2026-09-15

**Authors:** Ramachandra Reddy Gowda Venkatesha, Karthik Rajaram Mohan, Vasu Sridharrao, Saramma Mathew Fenn, Reethika Rathan

**Affiliations:** 1 Oral Medicine and Radiology, Vinayaka Mission's Sankarachariyar Dental College, Vinayaka Mission's Research Foundation (Deemed to be University), Salem, IND

**Keywords:** bone plate, mandibular fracture, non-union, osteomyelitis, pathological fracture

## Abstract

Mandibular fractures are a common occurrence, often associated with complex complications such as non-union and osteomyelitis. Non-union, the failure of a fracture to heal, can be particularly challenging in the mandible due to the limited soft tissue coverage and compromised blood supply in atrophic areas. Additionally, the presence of osteomyelitis, a bone infection, further complicates the healing process and increases the risk of implant failure. This case report aims to provide an overview of the management strategies for non-union of bone plating with mandibular fracture associated with osteomyelitis. The literature suggests that a multifaceted approach, including aggressive debridement, appropriate antibiotic therapy, and specialized fixation techniques, is often necessary to address these complex cases.

## Introduction

Mandibular fractures, particularly those occurring in the parasymphyseal region, are a common occurrence. The increase in the number of road traffic accidents has led to a substantial increase in fractures involving the maxillofacial region. Studies have reported an incidence rate of 25%-35% for mandibular parasymphyseal fractures, making them one of the most prevalent types of mandibular fractures [1]. The management of non-union in these fractures with plating presents a unique challenge, as the absence of teeth and poor bone quality in atrophic areas can significantly hinder the healing process. The single blood supply, with no collaterals, thick cortical plate, and less marrow space in the mandible contributes to the spread of infection, resulting in osteomyelitis. Trauma, infection, and an immunocompromised state, such as uncontrolled diabetes, increase the risk of osteomyelitis [2,3]. To address this issue, various management modalities have been explored, each with their own advantages and disadvantages.

## Case presentation

A 54-year-old male reported with a chief complaint of pain in his left lower jaw region for the past two months. The pain was chronic in onset, dull, intermittent, non-radiating in character, aggravated during mastication of food substances, and subsided for only two hours after intake of analgesic medication. His past surgical history revealed that he underwent bone plating for a mandibular parasymphyseal fracture one year ago, following a road traffic accident. The patient was treated with bone plating with lag screws for a left parasymphyseal fracture and a fracture of the right angle of the mandible. The patient gives no history of recent maxillofacial trauma but does give a history of spontaneous avulsion of his left mandibular first molar. A general examination revealed that his vitals were stable. Extraoral examination revealed a dimple on the left side of his face (Figure 1).

**Figure 1:**
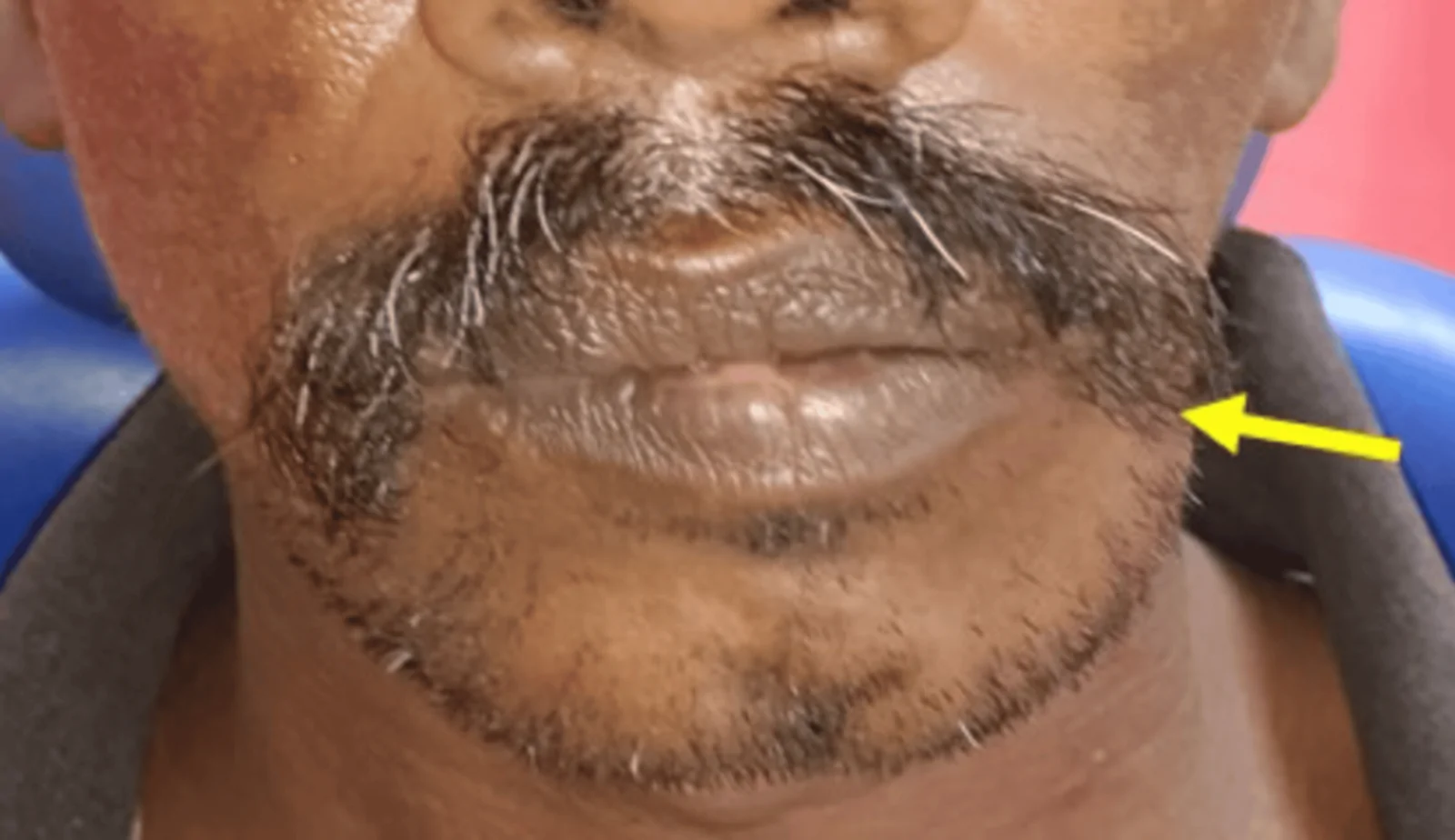
Extraoral examination revealed a dimple on the left side of the face (yellow arrow)

Intraoral examination revealed an intraoral swelling involving the buccal aspect of the attached gingiva, and exposure of bone plating in relation to the buccal vestibule, in the left mandibular second premolar tooth region (Figure 2).

**Figure 2:**
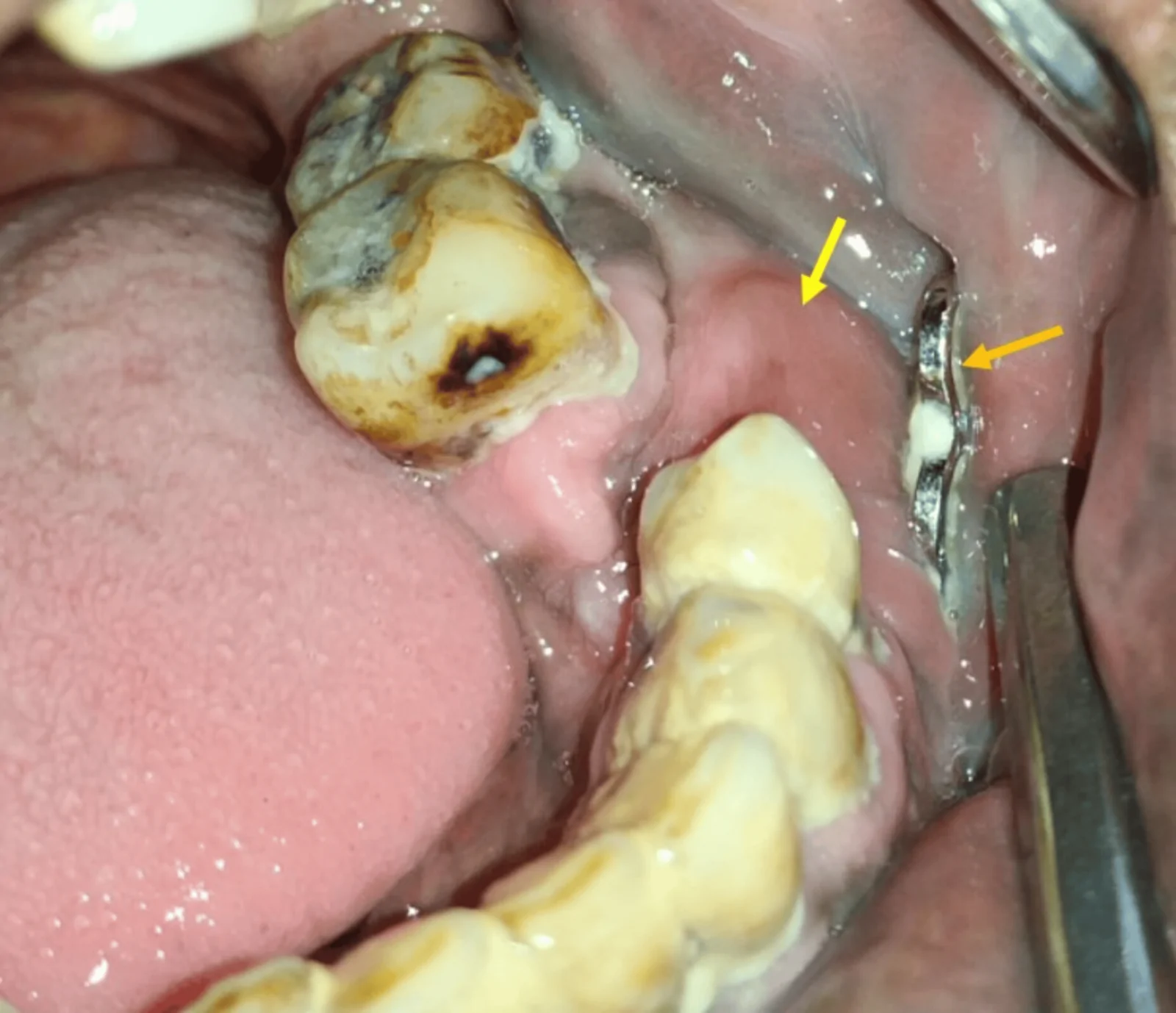
Intraoral examination revealed an intraoral swelling in relation to avulsed left mandibular second premolar region (yellow arrow) and exposure of bone plate (orange arrow)

The orthopantomography revealed a pathological fracture of the left parasymphyseal region of the mandible, with dislodged lag screws from bone plating in the mandible. The surrounding areas of the bone plate in the left parasymphyseal fracture region of the mandible also revealed a moth-eaten radiolucent appearance, suggestive of chronic osteomyelitis (Figure 3).

**Figure 3:**
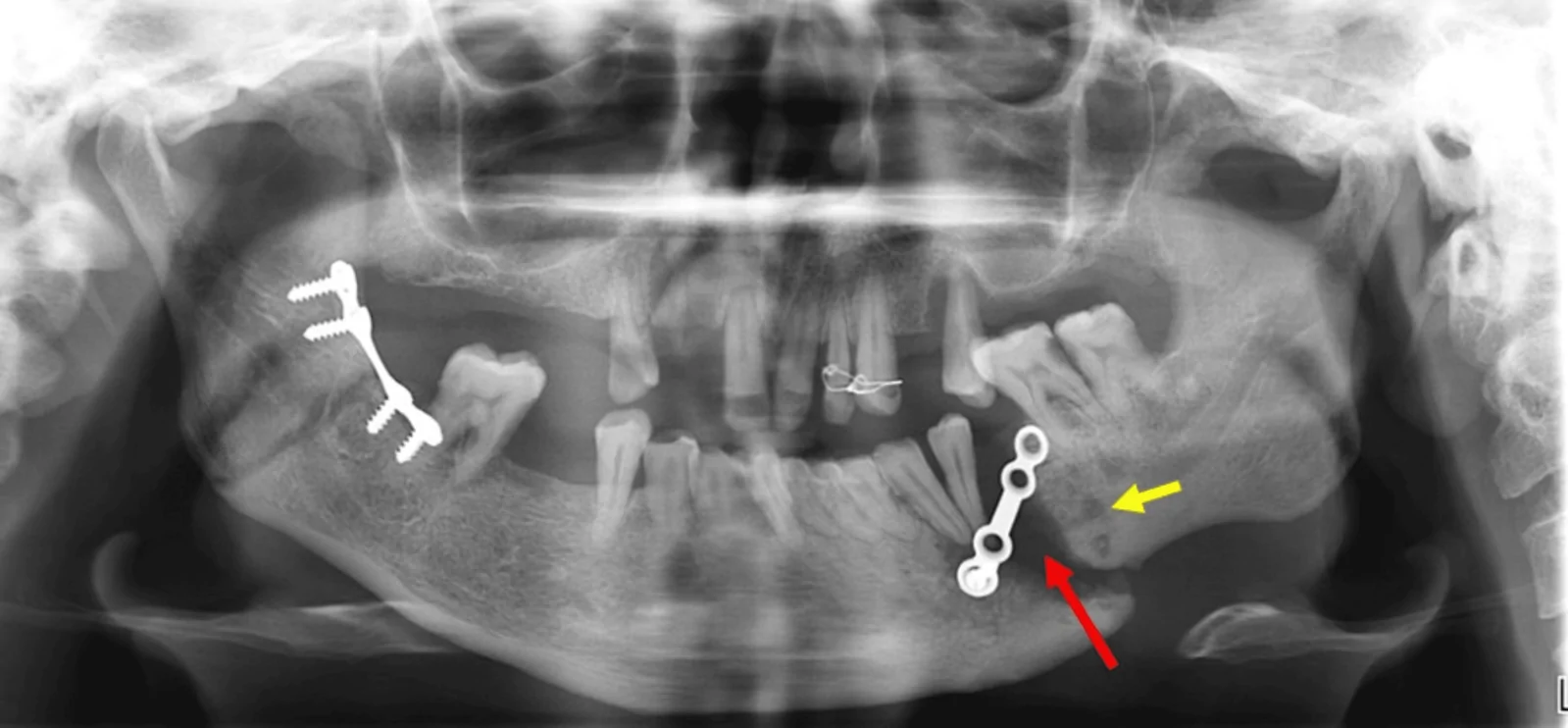
Orthopantomography revealed fracture of the left parasymphyseal region of mandible (red arrow), dislodged lag screws surrounded with moth-eaten radiolucent appearance (yellow arrow)

Correlating the above clinical and radiological features, a final diagnosis of non-union of bone plating in mandibular parasymphyseal fracture, associated with osteomyelitis, was made. His serological investigation revealed that his random blood glucose level was 258 mg/dL. The patient was deferred elective surgery due to his high blood glucose level. The patient was referred to a diabetologist for regulation of his blood glucose level and to the maxillofacial surgical unit for further surgical management.

## Discussion

Bone plating is a common surgical technique used to stabilize mandibular fractures, where a metal plate is fixed to the bone fragments to facilitate their alignment and promote healing. However, in cases of severe atrophic mandibular fractures, the reduced bone volume and poor blood supply in the affected areas can significantly increase the risk of non-union. The management of infected non-union mandibular fractures can be particularly challenging, as the presence of infection can further compromise the healing process and lead to additional complications, such as chronic osteomyelitis. Mandibular fractures are a common occurrence and often require surgical intervention for proper healing and restoration of function. One of the primary challenges in the management of these fractures is the risk of non-union, a condition where the fractured bone segments fail to heal properly. The use of bone plating, a widely adopted surgical technique, has been associated with non-union in certain cases, particularly in the presence of severe atrophy or infection [1].

In this research paper, we will explore the surgical techniques and management strategies employed in addressing the issue of non-union of bone plating in mandibular fractures.

Innovative approaches, such as computer-aided surgical simulation, have been explored to address these complex cases. These techniques involve the use of advanced imaging modalities and virtual planning software to optimize the surgical management of severely atrophic mandibular fractures, potentially reducing the risk of non-union and improving overall outcomes. Mandibular fractures are a common occurrence in the field of dentistry, and the management of these injuries has been a subject of ongoing research and debate. One of the significant challenges in the treatment of mandibular fractures is the potential for non-union, which can lead to various complications and negatively impact the patient's overall recovery [1]. Non-union of a surgically implanted bone plate is a rare but serious complication that can lead to persistent pain, disability, and potentially life-threatening complications, such as osteomyelitis, a severe bone infection [1-3]. In our case, poor oral hygiene, along with uncontrolled diabetes, has resulted in the occurrence of osteomyelitis and rejection of the bone plate.

Non-union, in the context of mandibular fractures, refers to the failure of the fractured bone segments to heal and reunite, resulting in a persistent separation between the fragments. This condition can be attributed to various factors, including the severity of the initial injury, the presence of infection, the adequacy of the surgical fixation, and the patient's underlying health status [1-3]. The mandible is subject to opposing forces of compression and tension at all times, which are regulated by loading and muscle attachments. These forces are equal while at rest. Although this is oversimplified, a fracture is typically brought together by forces of compression and separated by forces of tension. Fractures typically mend more quickly and are more resistant to separation when compressed. However, over-compression might jeopardize optimal bone healing and result in non-union if tension forces are not addressed [2]. According to studies, there is compression along the inferior border of the mandible and tension along the alveolar border in the mandibular region. Torsional, or rotational, forces are introduced, causing these two opposing forces to become mixed or even inverted as they move toward the symphysis and parasymphysis [3]. Minimizing the likelihood of infection consequences requires a thorough and prompt diagnosis of mandibular fractures. Infected mandibular fractures can be successfully treated with both intraoral and extraoral methods; however, patient selection should be done carefully, and the danger of wound dehiscence associated with the intraoral approach should be considered. Non-union is an uncommon consequence of infected mandibular fractures, which often heal without bone grafting. Nearly one-third of patients experience less serious surgical site problems, which can take up to three or four months to manifest. This highlights the significance of postoperative follow-up. In this patient population, thorough treatment during the primary phase is essential due to limited treatment compliance and a significant risk of surgical site complications [4]. The best outcomes are obtained when the monocortical external oblique miniplate is used. Stability at the optimal anatomical position is essential for the successful treatment of mandibular fractures because aberrant mobility at the fracture site can result in infection, malocclusion, and non-union [5].

The management of non-union of bone plating in mandibular fractures often requires a multifaceted approach, involving a combination of surgical techniques, targeted antibiotic therapy, and careful monitoring of the patient's progress [5]. The management of non-union in mandibular parasymphyseal fractures with plating can be challenging due to the absence of teeth and poor bone quality in atrophic areas, which can hinder the healing process [1]. Various surgical techniques for fracture management in the mandible are depicted in Figure 4.

**Figure 4:**
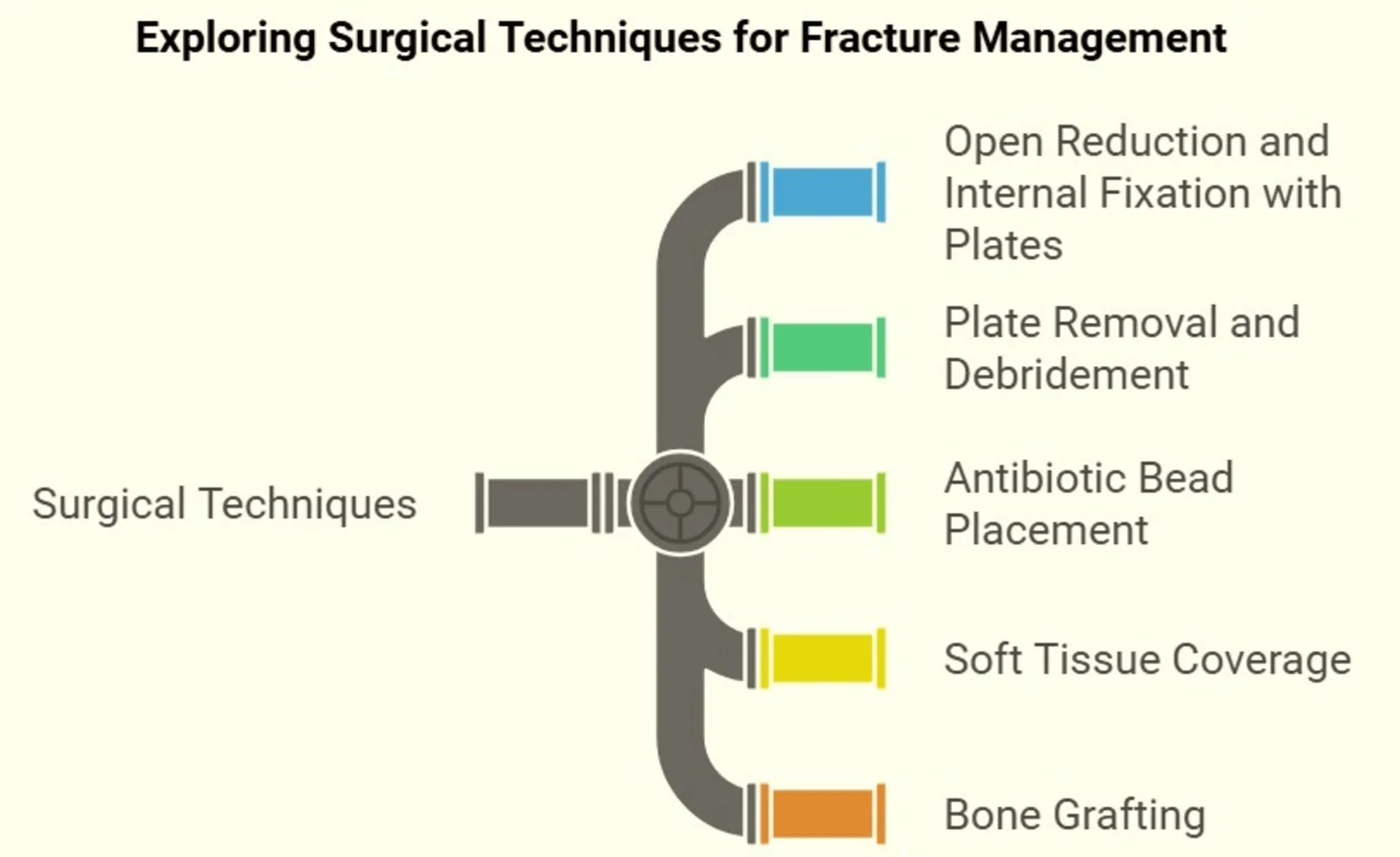
Surgical techniques for fracture management Image credit: Dr. Karthik Rajaram Mohan This image is licensed under Creative Commons Attributions License 4.0 International.

The advantages and disadvantages of surgical techniques in the management modalities of non-union of bone plating in mandibular fractures with osteomyelitis are enumerated (Table 1).

**Table 1:** Management modalities of non-union bone plating in mandibular fracture with osteomyelitis

Technique	Advantages	Disadvantages
Open reduction and internal fixation with plates	Provides rigid fixation, allowing for early mobilization and improved fracture healing	May be limited by poor bone quality, increasing the risk of bone-plate failure and non-union
Plate removal and debridement	Removes infected hardware and necrotic bone, promoting a healthier environment for healing	May compromise fracture stability, increasing the risk of further non-union
Antibiotic bead placement	Allows for targeted local antibiotic delivery to control infection	Requires additional surgery for bead removal, prolonging treatment
Soft tissue coverage	Improves vascularity and wound healing	Limited by the extent of soft tissue damage, may not adequately address underlying bone infection
Bone grafting	Enhances bony union by providing a scaffold for new bone formation	Increased surgical complexity, increased operating time, potential for graft resorption or infection

Newer, innovative techniques in the management of non-union bone plating in mandibular fractures have been explored in recent years. One such approach is the use of computer-aided surgical simulation, which has been shown to be effective in the treatment of severe atrophic mandibular fractures. This technique allows for precise pre-operative planning and the fabrication of customized fixation devices, which can improve the stability and alignment of the fracture site, thereby reducing the risk of non-union [1].

The various steps in computer-aided simulation in mandibular fracture management include the following: three-dimensional (3D) imaging of the fractured mandible, often using cone-beam computed tomography; precise pre-operative planning using specialized software to design custom fixation devices and simulate the surgical procedure; fabrication of the customized fixation devices, such as patient-specific plates or patient-specific distraction devices; intraoperative use of the computer-aided devices to achieve accurate reduction and fixation of the fracture; and postoperative monitoring and assessment of bone healing and restoration of function [1].

Computer-aided surgical simulation in mandibular fracture management has been reported to provide several benefits, including improved visualization of the fracture site, better alignment of the bone fragments, and more precise placement of fixation devices. This approach is particularly advantageous in cases of severe alveolar bone loss, where traditional fixation methods may be less effective due to poor bone quality and blood supply [1,2].

Management of non-union of bone plating in mandibular fracture cases often requires a multifaceted approach. In addition to surgical intervention, the use of local and systemic antibiotics, wound debridement, and temporary stabilization of the fracture site are crucial steps in the eradication of infection and the promotion of bone healing. The use of external fixation devices or internal fixation, combined with bone grafting, has also been reported as an effective strategy in the management of non-union of bone plating in mandibular fractures. These techniques aim to provide stable fixation, promote bone regeneration, and address any associated soft tissue defects. Patient-specific distraction devices have emerged as an effective strategy in the management of non-union of bone plating in mandibular fractures. These customized devices are designed based on the patient's specific anatomy and fracture characteristics, aiming to provide stable fixation and promote bone regeneration. The use of the patient-specific distraction device process is depicted in Figure 5 [1].

**Figure 5:**
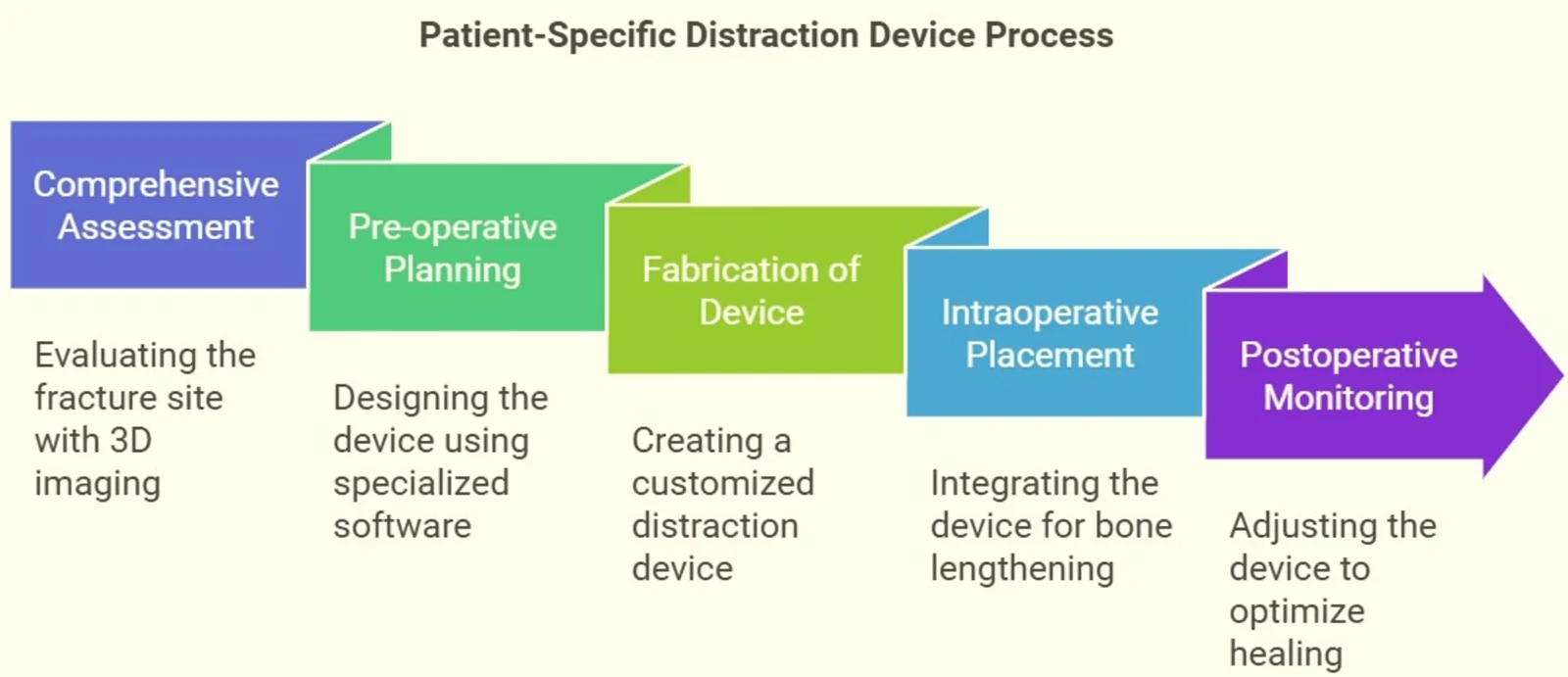
Patient-specific distraction device process Image credit: Dr. Karthik Rajaram Mohan This image is licensed under Creative Commons Attributions License 4.0 International.

By employing patient-specific distraction devices, surgeons can address the unique challenges posed by non-union of bone plating in mandibular fractures, such as severe atrophy or infection. These customized solutions can improve fracture stability, promote bone regeneration, and ultimately enhance the likelihood of successful healing and functional recovery for the patient [1].

Mandibular fractures are a common occurrence, often requiring surgical intervention to restore function and proper alignment. One of the significant complications associated with the management of mandibular fractures is the development of non-union, where the fractured segments fail to heal and fuse properly. In recent years, the use of patient-specific distraction devices has emerged as an effective strategy for addressing this challenge [1].

Distraction osteogenesis, a process of gradual bone lengthening, has been successfully employed in the management of non-union in mandibular fractures. This technique not only lengthens the hypoplastic mandible but also adds considerable bulk, which can be particularly beneficial in cases of severe atrophy. The introduction of computer-aided surgical simulation has further enhanced the precision and predictability of these distraction devices, allowing for customized solutions tailored to the individual patient's anatomy and needs [1].

Proper alignment and stabilization of the fractured segments are critical for successful bone healing. Conventional techniques, such as the use of splints and dentures, may not provide adequate stability, leading to fibrous union or non-union [2]. Distraction osteogenesis, on the other hand, offers a more stable and controlled environment for bone regeneration, minimizing the risk of non-union [1,2].

The management of infected non-union, a particularly challenging scenario, also benefits from the incorporation of distraction osteogenesis. The ability to gradually distract the bone, while simultaneously addressing the infection through aggressive debridement and targeted antibiotic therapy, can significantly improve the chances of successful fracture healing [3].

The existing literature provides ample evidence supporting the effectiveness of patient-specific distraction devices in the management of non-union in mandibular fractures. The techniques have demonstrated improved bone healing, reduced rates of complications, and enhanced functional outcomes for patients. In conclusion, the emergence of computer-simulated stabilization devices and patient-specific distraction devices for mandibular fractures has revolutionized the management of non-union in mandibular fractures. The most common location of osteomyelitis in the mandible is the mandibular angle [3].

The morphological deviations increase with the mandibular deviation. Therefore, the preferred treatment for adult patients with mandibular fractures is open reduction with fixation (ORIF) [4]. Surgical treatment is indicated for malocclusion, severe mandibular dislocation or luxation, and functional impairment. In conclusion, one of the various types of conventional osteotomies can be performed to treat mandibular malposition after malunited fractures [5]. By inserting 2 mm plates in the parasymphysis region and 2.5 mm plates in the mandibular angle, we were able to achieve normal occlusion, correct mandibular position, and facial morphology harmonization in our instances, without the need for bone or alloplastic grafts [6]. For prolonged comminuted mandibular fractures, ORIF ought to be the preferred course of treatment. For better results and a comparatively quicker return to function, a mandibular reconstructive plate can be utilized. As seen in this instance, proper debridement and plate removal can produce positive outcomes, even though infection is common in mandibular fractures [7]. The ORIF provides more satisfactory results than closed fixation of mandibular fractures [8]. Fathima et al. stated that both 3D standard and locking miniplates provide satisfactory results in the healing of mandibular fractures [9]. The 3D fixation plates increase the operating time in the fixation of mandibular angle fractures but provide satisfactory results, as standard fixation plates [10].

## Conclusions

In conclusion, the non-union of bone plating with mandibular fracture associated with osteomyelitis presents a significant challenge in management. A comprehensive multidisciplinary approach is essential in the management of such patients, involving aggressive debridement, antibiotic sensitivity tests, appropriate antibiotic therapy, and specialized fixation techniques. These are often necessary to address these complex cases and promote successful healing. Careful patient evaluation, early diagnosis, and a multidisciplinary treatment plan are crucial for achieving positive outcomes and increasing the quality of life in such patients.
